# Pulse actinomycin D as first-line treatment of low-risk post-molar non-choriocarcinoma gestational trophoblastic neoplasia

**DOI:** 10.1186/s12885-018-4512-5

**Published:** 2018-05-23

**Authors:** Lei Li, Xirun Wan, Fengzhi Feng, Tong Ren, Junjun Yang, Jun Zhao, Fang Jiang, Yang Xiang

**Affiliations:** Department of Obstetrics and Gynecology, Peking Union Medical College Hospital, Peking Union Medical College, Shuaifuyuan No.1, Dongcheng District, Beijing, 100730 China

**Keywords:** Actinomycin D, Gestational trophoblastic neoplasia, Transvaginal ultrasound, β-hCG

## Abstract

**Background:**

Little data exists predicting the resistance to actinomycin D (Act-D) single-agent for gestational trophoblastic neoplasia (GTN). The objective was to determine the overall success of pulse Act-D and the factors predictive of resistance to pulse Act-D in the treatment of low-risk, non-choriocarcinoma post-molar GTN.

**Methods:**

From January 2013 to October 2016, according to the FIGO criteria for the diagnosis of post-molar disease and the FIGO risk-factor scoring system for GTN, a total of 135 patients with post-molar non-choriocarcinoma GTN who were chemotherapy-naive with a FIGO score < 7 were treated with single-agent pulse Act-D as a first-line regimen, in Peking Union Medical College Hospital. The pulse Act-D regimen is defined as 1.25 mg/m^2^ (max 2 mg) IV push every other week. All patients were followed until May 2017. Epidemiological and clinical data were compared between patients with remission and resistance to Act-D to determine predictive factors by univariate and multivariate analysis.

**Results:**

Ninety-six of 135 patients (71.1%) achieved complete remission after first-line chemotherapy of pulse Act-D. In multivariate analysis, existing invasive uterine lesions observed by pre-chemotherapy transvaginal ultrasound (odds ratio [OR] 7.5, 95% confidence intervals [CI] 2.7–20.8), FIGO score ≥ 5 (OR 15.2, 95% CI 1.5–156.1) and pre-chemotherapy levels of β-hCG ≥ 4000 IU/L (OR 3.1, 95% CI 1.2–8.3) were independent high-risk factors predicting resistance to pulse Act-D as single-agent chemotherapy. During follow-up, no relapse, treatment-associated serious adverse events, or death occurred.

**Conclusions:**

As first-line chemotherapy, pulse Act-D was effective and tolerable for patients with low-risk post-molar non-choriocarcinoma. Existing invasive uterine lesions observed by pre-chemotherapy transvaginal ultrasound, a FIGO score ≥ 5, and pre-chemotherapy levels of β-hCG ≥ 4000 IU/L were independent factors for resistance to pulse Act-D.

**Electronic supplementary material:**

The online version of this article (10.1186/s12885-018-4512-5) contains supplementary material, which is available to authorized users.

## Background

At the initiation of treatment for gestational trophoblastic neoplasia (GTN) following a molar pregnancy, 94% of patients are International Federation of Gynecology and Obstetrics (FIGO) risk score 0 to 6, considered low risk and highly curable [1]. In the setting of a formal follow-up program, the expected cure rate for GTN after a molar pregnancy should be 100%, and the post-treatment relapse rate is 3% [1]. Although it has been reported that low-risk GTN is reliably and rapidly cured with combined methotrexate–dactinomycin and the toxicity is modest [2], single-agent chemotherapy is recommended as first-line treatment. Prompt diagnosis and treatment could limit the exposure of most patients to combination chemotherapy. There are several effective single-agent regimens; however, the choice of both the agents and dosage are individualized, taking several factors into consideration, such as effectiveness in reproductive-age women, cost efficiency, administration route, toxicity, possibility of exposure to second-line regimens, and the patient’s preference and adherence to treatment [3–6]. Methotrexate (MTX) and actinomycin D (Act-D) remain the most popular single-agent regimens for low-risk GTN. Three randomized clinical trials compared weekly MTX with biweekly Act-D, and the primary complete response rates were significantly lower for weekly MTX (49–53%) than for pulsed Act-D (69–90%) [3, 7, 8]. In a Cochrane database systematic review of 2016, the authors concluded that Act-D is probably more likely to achieve a primary cure in women with low-risk GTN, and less likely to result in treatment failure than a MTX regimen [9]. Based on these evidences, in Peking Union Medical College Hospital (PUMCH), we have adopted pulse intravenous Act-D as the first choice for low-risk non-choriocarcinoma GTN.

In a phase III trial, both biweekly Act-D regimen and weekly intramuscular MTX regimen were less effective if the FIGO risk score was 5 or 6, or if the diagnosis was choriocarcinoma [3]. Apart from this study, there is no other evidence available regarding the predictors for drug resistance of Act-D. In our retrospective analysis, we tried to find whether there were definite factors predicting resistance to Act-D for low-risk post-molar non-choriocarcinoma GTN.

## Methods

This is a retrospective study performed in a tertiary teaching hospital (PUMCH). The Institutional Review Board of PUMCH has approved this study. The clinical registration number is NCT03291275 (ClinicalTrials.gov, retrospectively registered on September 25, 2017, and a prospective study is being carried out also). All post-molar GTN patients treated with a single-agent as first-line chemotherapy from January 2013 to October 2016 were recruited and analyzed. Informed consent was obtained from participants to be analyzed in this study. Pretreatment evaluations included computed tomography (CT) scans of the lung, transvaginal ultrasound (TVS), and serial β-hCG assays. Diagnosis was confirmed according to the 2002 FIGO criteria of post-molar disease, a combined anatomic staging and modified FIGO/WHO risk-factor scoring system for GTN [10]. The factors needed to diagnose post-molar GTN included at least one of the following: (1) β-hCG plateau for 4 consecutive values over 3 weeks; (2) β-hCG rise of10% for 3 values over 2 weeks; (3) β-hCG persistence 6 months after molar evacuation; (4) histopathological diagnosis of invasive mole; or (5) presence of metastatic disease. In our study, the patients were excluded if (1) they were not treated with Act-D, or (2) the FIGO score was ≥7, or (3) pathological examination suggested choriocarcinoma, or (4) they had received prophylactic chemotherapy before definite diagnosis of GTN.

Act-D dosing was calculated based on actual body surface area as 1.25 mg/m^2^ intravenous dose every 2 weeks (maximum single dose no more than 2 mg). Remission was achieved when β-hCG levels reached and remained normal for at least three consecutive weeks. Resistance or failure to Act-D treatment was defined using the modified FIGO criteria, which included a β-hCG plateau of more or less 10% for four consecutive values over 3 weeks, or a rise in β-hCG level over 2 courses of Act-D treatment. Patients resistant to Act-D received combination regimens, including FAV (floxuridine, Act-D, and vincristine), FAEV (floxuridine, Act-D, etoposide, and vincristine), or EMA/CO (etoposide, MTX, Act-D, cyclophosphamide, and vincristine). After achieving a normal β-hCG level, one to three courses of consolidation therapy were administered according to metastatic status and FIGO score. All patients were followed until May 2017. After the last course of chemotherapy, levels of β-hCG were checked monthly for at least 1 year. Relapse was defined as increasing and abnormal β-hCG test results with or without invasive lesions after remission. All patients were interviewed by telephone call to ascertain their status due to GTN, and relapse was confirmed by tests and examinations in outpatient clinics.

Patients’ epidemiological data, pre-chemotherapy assessment (β-hCG assay, computed tomography scans, TVS), objective response, and survival outcomes were collected for analysis based on medical records, telephone review and outpatient clinic records. Pre-chemotherapy β-hCG was further classified into several categories (1000, 2000, 3000, 4000, 5000, 10,000, 50,000 and 100,000 IU/L) for further cut-off value analysis. Invasive uterine lesions in pre-treatment TVS were defined as uterine lesions with a maximum diameter of ≥5 mm appearing as abnormal areas of increased echogenicity within the myometrium with the presence of increased intra-tumor and peri-tumor blood flow compared with the adjacent tissue [11]. Data of adverse events were collected retrospectively and graded according to the Common Terminology Criteria for Adverse Events (CTCAE) v4.03 [12] from medical records and clinical follow-up.

The data of all patients were listed in Additional file 1.

### Statistical analysis

Statistical analyses were carried out using SPSS statistical software (version 19.0, SPSS Inc. Chicago, IL). Potential confounders were identified using the nonparametric κ^2^ test or Fisher’s exact test and Mann-Whitney *U* test. Multiple parameter analyses were performed using binary logistic analysis calculating odds ratios (OR) and 95% confidence intervals (95% CI). ROC curves were applied to calculate cut-off values of chemotherapy courses and uterine lesions predicting remission or failure to second-line therapy.

## Results

For 178 cases treated with single-agent chemotherapy, 135 eligible patients with post-molar GTN who were chemotherapy-naive were included in the study. Their median age was 28.5 years (range 17.9–53.7) and the median follow-up period was 20.5 months (range 8–63). Ninety-six of 135 patients (71.1%) achieved complete remission after first-line chemotherapy of Act-D, and 39 patients (28.9%) failed Act-D treatment. In univariate analysis between patients of remission and failure to Act-D, most of the epidemiological and clinical variables had no significant differences, except for following parameters: existing invasive uterine lesions observed by pre-chemotherapy TVS (*P* < 0.001), median diameter of invasive uterine lesions (*P* = 0.026), median FIGO score (*P* < 0.001), and median serum β-hCG before chemotherapy (*P* < 0.001) (Table 1). Among cut-off values of serum β-hCG, 4000 IU/L had maximal areas of ROC (0.729, 95% CI 0.630–0.828, *P* < 0.001).

**Table 1:** Patient and tumor characteristics

	Remission after Act-D treatment (*n* = 96)	Resistant to Act-D treatment (*n* = 39)	*P*
Median age (years) (range) at diagnosis	28.2 (17.9–53.7)	30.0 (18.2–51.6)	0.387
Median body weight (Kg) (range)	57 (40–88)	57 (44–91)	0.804
Median body height (cm) (range)	160.5 (150–170)	160 (150–170)	0.032
Median body mass index (kg/m^2^) (range)	21.8 (15.6–34.4)	21.9 (18.0–33.5)	0.561
Median parity (range)	2 (1–7)	2 (1–6)	0.180
Median gravidity (range)	0 (0–2)	1 (0–2)	0.086
Pathology of hydatid mole			0.425
Partial mole	27/91 (29.7%)	14/38 (36.8%)	
Complete mole	64/91 (70.3%)	24/38 (63.2%)	
Median numbers of curettage for hydatid mole (range)	2 (1–2)	2 (1–2)	0.665
Invasive uterine lesions			< 0.001
No	85 (88.5%)	17 (43.6%)	
Yes	11 (11.5%)	22 (56.4%)	
Median diameter of invasive uterine lesions (mm) (range)	21 (5–45) (*n* = 11)	32.5 (13–87) (*n* = 22)	0.026
FIGO staging			0.816
Stage I	39 (40.6%)	15 (38.5%)	
Stage III	57 (59.4%)	24 (61.5%)	
Median FIGO/WHO scores (range)	1 (0–6)	3 (0–6)	< 0.001
Median serum β-hCG before chemotherapy (IU/L) (range)	339 (60–46,940)	5517 (86–159,160)	< 0.001

In multivariate analysis, existing invasive uterine lesions observed by pre-chemotherapy TVS (OR 7.5, 95% CI 2.7–20.8, *P* < 0.001), a FIGO score ≥ 5 (OR 15.2, 95% CI 1.5–156.1, *P* = 0.022) and pre-chemotherapy β-hCG levels ≥4000 IU/L (OR 3.1, 95% CI 1.2–8.3, *P* = 0.021) were independent risk factors predicting failure of Act-D treatment. Compared with other β-hCG categories, the cut-off value of 4000 IU/L had the greatest significance in predicting resistance to Act-D in multivariate models. For patients with none of three risk factors, the remission rate after Act-D treatment was 92.4% (73/79); however for those with any of the aforementioned independent risk factors, the remission rate decreased to 41.1% (23/56); for those with one, two and three of independent risk factors, the remission rates were 50.0% (14/28), 33.3% (9/27) and 0 (0/1) respectively.

Pre-chemotherapy TVS showed that 33 patients (24.4%) had invasive uterine lesions. A comparison of patients with remission or failure to Act-D, indicated that none of the epidemiological and clinical variables (including hCG level and FIGO scores) were significantly different except for median diameters of lesions: 21 mm (range 5–45, 11 cases) vs 32.5 mm (range 13–87, 22 cases) respectively (*P* = 0.026). For patients with invasive uterine lesions < 25 mm and ≥ 25 mm, remission rates of the diameters after Act-D treatment were 60.0% (6/10) and 21.7% (5/23) respectively (*P* = 0.042).

For 39 patients resistant to Act-D, 6 cases (15.4%) were still resistant to second-line chemotherapy, of which 5 cases (83.3%) had invasive uterine lesions even before the treatment. These 6 patients achieved remission after third-line chemotherapy. Resistance to Act-D was associated with more courses of chemotherapy (*P* < 0.001), longer treatment duration (*P* < 0.001) but similar recurrence-free survival (*P* = 0.198).

There were no serious (grade 3/4/5) adverse events regarding the treatment with Act-D. The most common grade 2 adverse events were nausea (20 cases, 14.8%), constitutional symptoms (12 cases, 8.9%), alopecia (10 cases, 7.4%), neutropenia (10 cases, 7.4%), vomiting (10 cases, 7.4%), other gastrointestinal tract symptoms (8 cases, 5.9%), and anemia (6 cases, 4.4%). No extravasation happened in our study.

During follow-up, all the patients survived without relapse. After complete remission of β-hCG levels, 6 patients (4.4%) volunteered to undergo hysterectomy.

## Discussion

We selected Act-D as first-line for low-risk GTN based on the treatment effects and costs. As to the safety and effectiveness profiles of our data, Act-D has favorable remission rate with tolerable adverse events coincident with previous reports [13–17]. In a randomized trial of the Gynecologic Oncology Study Group, the biweekly Act-D regimen had a higher complete response rate than the weekly intramuscular MTX regimen in low-risk GTN (70% versus 53%, *P* = 0.01) [3]. Act-D may be associated with a greater risk of severe adverse events than a MTX regimen, and Act-D will produce local tissue injury if intravenous extravasation occurs [18], which did not occur in our study. Nevertheless, there may be little or no difference between the pulsed Act-D regimen and the MTX regimen with regard to side-effects [9]. However, in a retrospective study, Uberti et al. [19] discovered that first-line side effects frequency were high but intensity was low; stomatitis was higher with MTX/folinic acid (*p* < 0.01) and nausea and vomiting with Act-D (*p* < 0.01). In their report, nausea and vomit happened in 59.6% patients, but the severity (grading) was unclear. In our study, severe alopecia, nausea and vomiting with Act-D had higher frequency than MTX reported by others [3, 20], which deserves caution for the patient education and counseling. Initial treatment with MTX-folinic acid is slightly less expensive, but needs to be given for a longer time to achieve remission. Therefore, treatment costs of low-risk GTN are almost equal between Act-D and MTX-folinic acid but with different time to remission. The incremental cost effectiveness ratio of initial treatment with Act-D over MTX-folinic acid is $1.07 USD/day of earlier treatment completion [20]. However, the analysis of cost-effectiveness was not included in our study. We must be very cautious for the extrapolation of these data in Chinese population.

Few reports described predictors for the resistance to first-line monotherapy regimens, and most available evidence focused primarily on the treatment of MTX. These predictors included β-hCG, FIGO scores, pathologic categories of GTN, tumor sizes, interval durations from the index pregnancy, and imaging findings et al. In this study of a large cohort patient, β-hCG, FIGO scores and TVS discoveries were independent factors of resistance to Act-D. These findings not only confirmed to previous reports, but also presented new evidence of response to first-line therapy.

The role of β-hCG in predicting resistance to Act-D is not very clear. Pre-treatment β-hCG levels of > 100,000 IU/L may have high rates of resistance to multiple doses of intramuscular MTX [21]. It is proposed low-risk post-molar GTN patients with β-hCG > 400,000 IU/l may benefit from multi-agent chemotherapy [22]. In a cohort of low-risk GTN patients, You et al. found choriocarcinoma pathology and calculated that individual β-hCG clearance ≤0.37 I/day was a major independent predictive factor for MTX resistance risk [23]. Later, they developed hCGres, a modeled kinetic parameter calculated after 3 full-dose MTX cycles, which gave a reproducible value for identifying patients with MTX resistance [24]. Our study, for the first time, provided a β-hCG cut-off value defining 4000 IU/ml for Act-D as a first-line regimen in low-risk non-choriocarcinoma GTN patients. For patients with β-hCG < 4000 IU/ml, the probability of failure to first-line therapy would increase by more than three folds, which warrants more rigorous evaluation in prospective studies in order to exclude bias from selection or sample size.

There are few data about the association of FIGO score and resistance to Act-D. Existing low-risk and high-risk discrimination according the FIGO scoring system is probably not very sensitive for predicting resistance to single-agent regimen of MTX. For patients treated with single-agent MTX, the primary cure rate ranged from 75% for those with a FIGO score of 0–1 to 31% for those with a FIGO score of 6 [1]. In a prospective study of MTX for low-risk GTN, authors suggested that cases with a FIGO score ≥ 6 should be considered high risk for MTX treatment [25]. In a retrospective study applying 4 doses of intramuscular MTX, patients with low risk GTN who had a FIGO score of 6 had high rates of resistance to the regimen and required further treatment [21]. According to data of Gilani et al., a lower mean FIGO score predicts a better outcome for the regimen of weekly intramuscular MTX as first line therapy [26]. Lurain et al. [16] found resistance to sequential MTX and Act-D chemotherapy was significantly associated with an original FIGO score ≥ 3. In the study of Chapman-Davis et al. [27], patients with FIGO score 0–2, 3–4 and 5–6 had complete response rates of 87, 68 and 52% (*P* < 0.0001). In our study, we found a FIGO score ≥ 5 denoted a higher rate of resistance to Act-D. Based on these results, a more appropriate discrimination of the FIGO score system could be applied for the purpose of evaluating treatment effects of single-agent of MTX or Act-D.

Correlations between ultrasound findings and monotherapy effects were neglected by most researchers, and even fewer studies explored the correlation of ultrasound finding and resistance to Act-D. Some authors considered whether to incorporate the uterine artery pulsatility index (UAPI) into the FIGO scoring system so that patients with a score of 6 and a UAPI≤1 might be upstaged and therefore offered combination chemotherapy rather than MTX [28]. Among patients who are candidates for second-line treatment on the basis of β-hCG levels, ultrasound response to chemotherapy (defined as decreased myometrial lesions in echogenicity), Doppler signal, or size could identify those in whom further MTX administration could induce a delayed complete response [11]. In our study, we first report existing invasive uterine lesions and their maximum diameter in TVS had the most effective prediction effects for resistance to Act-D. This discovery could help decision-making in discussions with patients regarding the selection of chemotherapy plans.

Limitations of this study include its retrospective nature which is associated with selection bias and recall bias, and the limited sample sizes of patients restrict further generalization and extrapolation of our findings, especially about the cut-off values of β-hCG and FIGO score for predicting resistance to Act-D.

## Conclusion

Our practice of employing single-agent Act-D as first-line therapy for low-risk non-choriocarcinoma post-molar GTN has achieved a favorable remission rate with tolerable adverse events. In a multivariate model, existing invasive uterine lesions in pre-chemotherapy TVS, FIGO score ≥ 5, and pre-chemotherapy β-hCG ≥ 4000 IU/L were independent factors for resistance to Act-D. Resistance to Act-D was associated with more courses of chemotherapy and longer treatment duration but similar recurrence-free survival compared with remission after Act-D treatment. These data will provide practical evidence for discussions with patients with GTN about prognosis and the selection of specific chemotherapy plans.

## Additional file

Additional file 1: Raw data. This file contains data generated or analysed during this study. (XLSX 24 kb)

## Abbreviations

Act-D: Actinomycin D
CI: Confidence intervals
CT: Computed tomography
CTCAE: Common terminology criteria for adverse events
EMA/CO: Etoposide, MTX, Act-D, cyclophosphamide, and vincristine
FAEV: Floxuridine, Act-D, etoposide, and vincristine
FAV: Floxuridine, Act-D, and vincristine
FIGO: International Federation of Gynecology and Obstetrics
GTN: Gestational trophoblastic neoplasia
MTX: Methotrexate
OR: Odds ratio
PUMCH: Peking Union Medical College Hospital
TVS: Transvaginal ultrasound
UAPI: Uterine artery pulsatility index
